# Chemokine-Receptor Modulation Shapes Neuroinflammatory and Biomechanical Responses in Organotypic Hippocampal Cultures After Oxygen-Glucose Deprivation

**DOI:** 10.1021/acschemneuro.6c00005

**Published:** 2026-05-15

**Authors:** Natalia Bryniarska-Kubiak, Andrzej Kubiak, Ewa Trojan, Alessandro Podestà, Małgorzata Lekka, Agnieszka Basta-Kaim

**Affiliations:** † Laboratory of Immunoendocrinology, Department of Experimental Neuroendocrinology, 69714 Maj Institute of Pharmacology, Polish Academy of Sciences, 12 Smętna St., 31-343 Kraków, Poland; ‡ Eli and Edythe Broad CIRM Center for Regenerative Medicine and Stem Cell Research, Keck School of Medicine, 12223 University of Southern California, Los Angeles, California 90033, United States; § Department of Biophysical Microstructures, Institute of Nuclear Physics, Polish Academy of Sciences, 152 Radzikowskiego St., 31-342 Kraków, Poland; ∥ Department of Physics “Aldo Pontremoli”, 9304 Università degli Studi di Milano, via Celoria 16, 20133 Milano, Italy

**Keywords:** ischemic stroke, neuroinflammation, chemokines, organotypic hippocampal cultures, biomechanics

## Abstract

Ischemic stroke is a life-threatening neurological condition that frequently leads to severe brain damage and long-term disability. Current therapeutic options remain limited and are largely confined to anticoagulant-based interventions. Despite extensive evidence implicating inflammation in stroke pathogenesis, effective therapies targeting neuroinflammatory mechanisms are still missing. Given that chemokines and their receptors have been successfully targeted in clinical contexts outside stroke, we identified these signaling axes as promising candidates for therapeutic modulation in ischemic injury. We tested the hypothesis that pharmacological modulation of selected chemokine-receptor pathways alters the neuroinflammatory response and tissue viability following ischemia. Using organotypic hippocampal cultures subjected to oxygen-glucose deprivation, we demonstrate that inhibition of CX3CR1, CCR2, and CXCR4 differentially affects tissue viability under ischemic conditions. Owing to the pronounced biological effects observed upon CCR2 inhibition, we further investigated its mechanistic basis using atomic force microscopy. These analyses revealed that Irbesartan treatment is associated with cytoskeletal reorganization and significant alterations in the mechanical properties of ischemic brain tissue. In summary, our study establishes the organotypic hippocampal culture-oxygen-glucose deprivation model as a robust platform for investigating neuroinflammatory processes in ischemic stroke. We identify key chemokine-receptor circuits that modulate ischemic injury and uncover a previously underappreciated contribution of tissue biomechanics to the pathophysiological response to ischemia.

## Graphical abstract

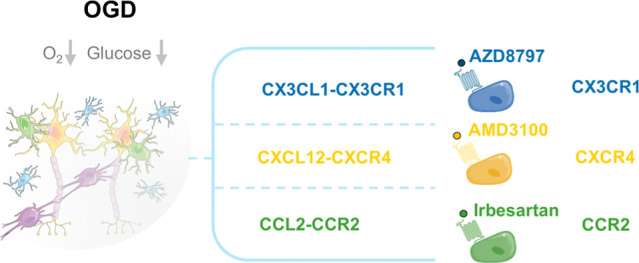

## Background

Ischemic stroke is a neurodegenerative condition caused by the occlusion of blood vessels, leading to an interruption of blood flow in the brain. It is a life-threatening event that often results in long-term disabilities among survivors. Understanding the interactions between molecules and cells that are activated during a stroke may reveal novel therapeutic targets for more effective treatment. Currently, clinical interventions are largely limited to anticoagulant therapy, aimed at dissolving clots and restoring blood flow. To be effective, recombinant tissue plasminogen activator (rt-PA, or alteplase) must be administered intravenously within a narrow therapeutic window.^1^ While this limited time frame alone poses a major challenge in stroke treatment, neuronal damage in the affected brain areas can continue to evolve over a longer period. Reperfusion and neuroinflammation are interrelated processes that contribute to subsequent injury of nervous tissue.^2^ Neuroinflammation is a complex response orchestrated by both resident brain cells and peripheral immune cells recruited to the ischemic site by chemotactic signaling.^3^ Among chemotactic molecules, chemokines have been extensively studied for their roles in immune system function,^4–6^ whereas their contribution to the pathophysiology of nervous system disorders remains incompletely defined. The expression of some chemokine receptors is cell-type-specific, making them promising therapeutic targets. Notably, CX3CR1, the receptor for CX3CL1—the sole member of the CX3C chemokine family—is selectively expressed on microglia,^7,8^ highlighting its importance in regulating neuroinflammatory responses. In addition, in silico studies have identified sartans as antagonists of CCR2. Sartans, commonly used antihypertensive agents, are particularly relevant in the context of stroke, as hypertension is a major risk factor for stroke.^9^ Thus, investigating Sartans potential role as CCR2 modulators in ischemic stroke may offer novel therapeutic insights. Another key chemokine, CXCL12, is constitutively expressed by both neurons and glial cells.^10^ It signals primarily through two receptors: CXCR4 and CXCR7. CXCR7 expression is low in the brain, while CXCR4 is widely expressed in the central nervous system and mediates most of CXCL12’s biological effects, making it a principal target for intervention.^11^ CXCR4 antagonist—AMD3100 (Plerixafor, marketed as Mozobil) is already clinically approved for the mobilization of hematopoietic stem cells, and its pharmacological profiles are well-established.^12^

Building on previous reports, we hypothesized that pharmacological targeting of chemokine receptors can uncover how specific chemokine-receptor axes contribute to neuroinflammatory responses in ischemia-reperfusion injury. To this end, we focused on three chemokine receptors—CX3CR1, CXCR4, and CCR2—and used their respective antagonists: AZD8797,^13^ AMD3100,^14^ and Irbesartan.^15^ We employed organotypic hippocampal cultures (OHCs) as an experimental model, which maintain the complex architecture of brain tissue and enable the study of cellular interactions.^16–18^ While brain architecture is largely preserved when using OHCs, this model allows the separation of brain tissue from the periphery, enabling precise targeting of neuroinflammatory processes. To date, only multicellular systems such as brain organoids allow for such experimental setups; however, while extremely insightful for neuroscientific research, they are still unable to fully recapitulate the physiological architecture of brain tissue.^19^ To mimic stroke conditions, OHCs were subjected to oxygen-glucose deprivation (OGD), a well-established in vitro model of ischemic stroke. OHCs neuroinflammatory response to OGD was tested in the presence of the chemokine receptor modulators. Moreover, recent studies suggest that changes in the mechanical properties of the microenvironment influence immune cell function^20–22^ and are linked to immune responses.^23,24^ Consequently, biophysical factors are increasingly recognized as contributors to stroke pathophysiology.^25,26^ In our recent work, we demonstrated that OHCs become stiffer following OGD.^16^ Therefore, in the current study, we also explored how CCR2 modulation affects the mechanical properties of OHCs.

Our findings highlight the important role of chemokines and their receptor circuits in the neuroinflammatory response of OHCs to OGD. The partially neuroprotective effect of CCR2 modulation by Irbesartan, also reflected in our nanomechanical analysis, may represent a promising strategy to mitigate the detrimental consequences of neuroinflammation following cerebral ischemia and reperfusion.

## Results

### Oxygen Glucose Deprivation Increases Cell Death and Neuronal Loss in Organotypic Hippocampal Cultures

In our study, organotypic hippocampal cultures (OHCs) were subjected to a 40 min oxygen-glucose deprivation (OGD) procedure. As previously reported,^16^ this resulted in a significant increase in cell death, confirmed by elevated lactate dehydrogenase (LDH) release in OHCs following OGD (Figure 1A). At the cellular level, we observed a pronounced decrease in the fluorescence intensity of the neuronal marker MAP-2 (Figure 1B; Supporting Information Figure S1A,B), indicating the high vulnerability of neuronal cells to OGD. Similarly, we observed a reduction in the signal from IBA1-positive cells (Supporting Information Figure S1C,D), indicating microglia were also partially depleted 24 h after OGD. Given our previous findings that OGD induces a robust inflammatory response and enhances the secretion of several proinflammatory cytokines in OHCs,^16^ we next aimed to characterize the expression of clinically relevant chemokines. We used ELISA to quantify CXCL12, CX3CL1, and CCL2 protein levels in OHCs. The analysis revealed a significant increase in both CX3CL1 (Figure 1C) and CCL2 (Figure 1D) expression following OGD, whereas CXCL12 levels remained unchanged (Figure 1E). Based on these results, we next asked to what extent the modulation of brain-specific receptors for these chemokines could influence neuroinflammatory responses and cell death in OHCs.

**Figure 1:**
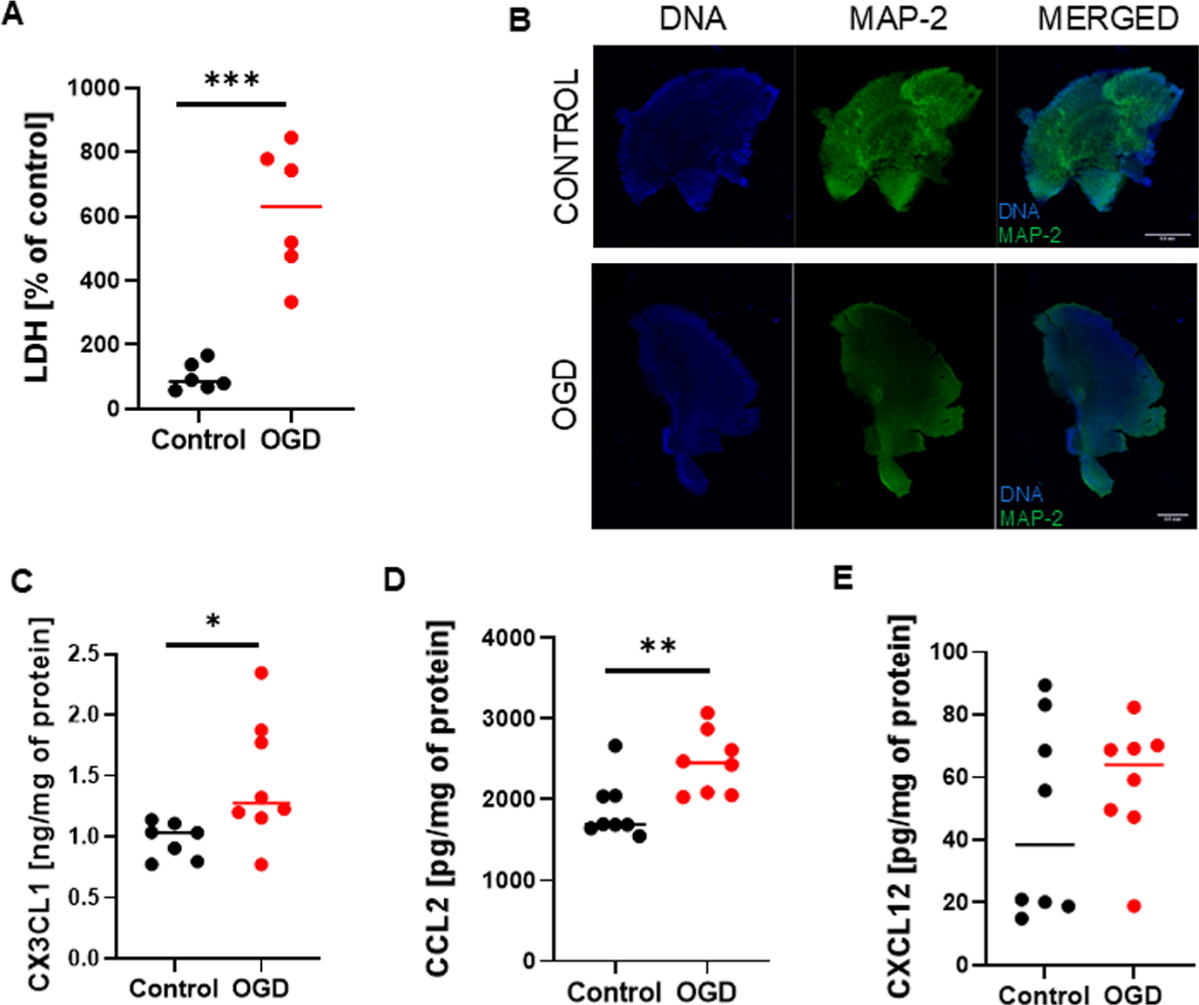
(A) Impact of OGD on LDH release by OHC. (B) Immunofluorescence staining of OHC, nuclei (blue—DNA), neurons (green—MAP-2), scale bar = 500 μm. Impact of OGD on expression levels of (C) CX3CL1, (D) CCL2, and (E) CXCL12.

### CX3CR1 Modulation and Microglia Depletion Affect Neuroinflammatory Status in OHC Undergoing OGD

Neurons are the primary source of CX3CL1 in the brain, although some studies suggest that under stress conditions, astrocytes may also contribute to its secretion.^27^ In our model, we demonstrated that OGD leads to a significant increase in CX3CL1 expression in OHCs (Figure 1C). Consequently, modulation of its receptor CX3CR1 using the selective antagonist AZD8797 showed a trend toward decreased LDH release in OGD-subjected OHCs, although it did not reach statistical significance (*t*-test, *p* = 0.1) (Figure 2A).

**Figure 2:**
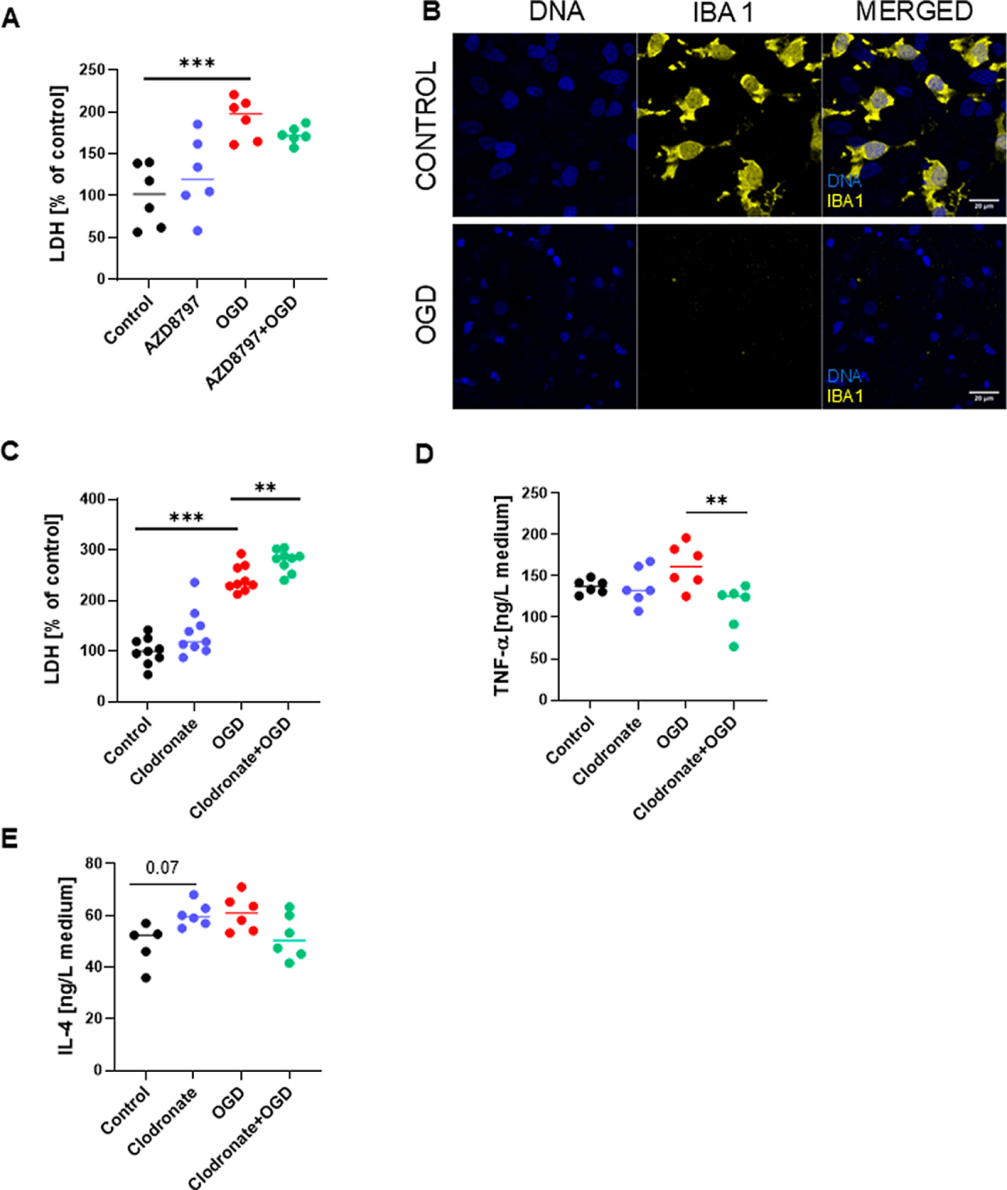
(A) Impact of OGD and CX3CR1 modulation by AZD8797 on LDH release by OHC. (B) Immunofluorescence staining of OHC, nuclei (blue—DNA), microglia (yellow—IBA1). (C) Impact of Clodronate and OGD on LDH release by OHC. Impact of Clodronate and OGD on expression of (D) TNF-α and (E) IL-4 by OHC.

CX3CR1 is specifically expressed on microglial cells.^28^ Thus, we next sought to investigate how microglial depletion affects OHC susceptibility to OGD. To this end, we treated OHCs with Clodronate, which effectively depleted microglia (Figure 2B). Microglial depletion resulted in a significant increase in LDH release following OGD, indicating enhanced tissue vulnerability to ischemic conditions (Figure 2C).

We then examined how microglial depletion affects the expression of key cytokines involved in neuroinflammation, specifically TNF-α and IL-4. In control (non-OGD) OHCs, Clodronate treatment did not significantly alter TNF-α expression (Figure 2D). However, in OGD-subjected OHCs, microglial depletion led to a significant decrease in TNF-α expression (Figure 2D). Interestingly, IL-4 expression increased following Clodronate treatment in control and OGD-treated OHCs (Figure 2E). In contrast, IL-4 expression in Clodronate-treated OHCs subjected to OGD remained at levels comparable to untreated controls (Figure 2E). In summary, while modulation of the CX3CL1-CX3CR1 (neuron-microglia) axis did not significantly affect the OHC response to OGD (Figure 2A,B), depletion of microglia disrupted the inflammatory response of OHCs, highlighting the complex regulatory role of microglia in ischemia-induced neuroinflammation (Figure 2B–E).

### Modulation of CXCR4 with AMD3100 Affects IL-18 Secretion

AMD3100, known as Plerixafor, is a drug used in clinical practice to mobilize hematopoietic stem cells for transplantation purposes.^29^ Its well-characterized pharmacological profile makes AMD3100 a potentially promising candidate for novel clinical applications. In our study, AMD3100-treated OHCs exhibited an elevated level of LDH release following OGD; however, this increase did not reach statistical significance (*p* = 0.09) (Figure 3A). AMD3100 treatment did not affect CXCL12 expression in either untreated OHCs or those subjected to OGD (Figure 3B). Similarly, AMD3100 had no significant effect on IL-4 (Figure 3C), IL-6 (Figure 3D), or IL-10 expression (Figure 3E). In contrast, IL-18 expression was increased in OGD-subjected OHCs following AMD3100 treatment, whereas no change was observed in control OHCs (Figure 3F). These results suggest that CXCR4 may modulate specific inflammatory pathways, such as IL-18 upregulation, although its role in maintaining immune homeostasis under ischemic conditions remains to be fully elucidated.

**Figure 3:**
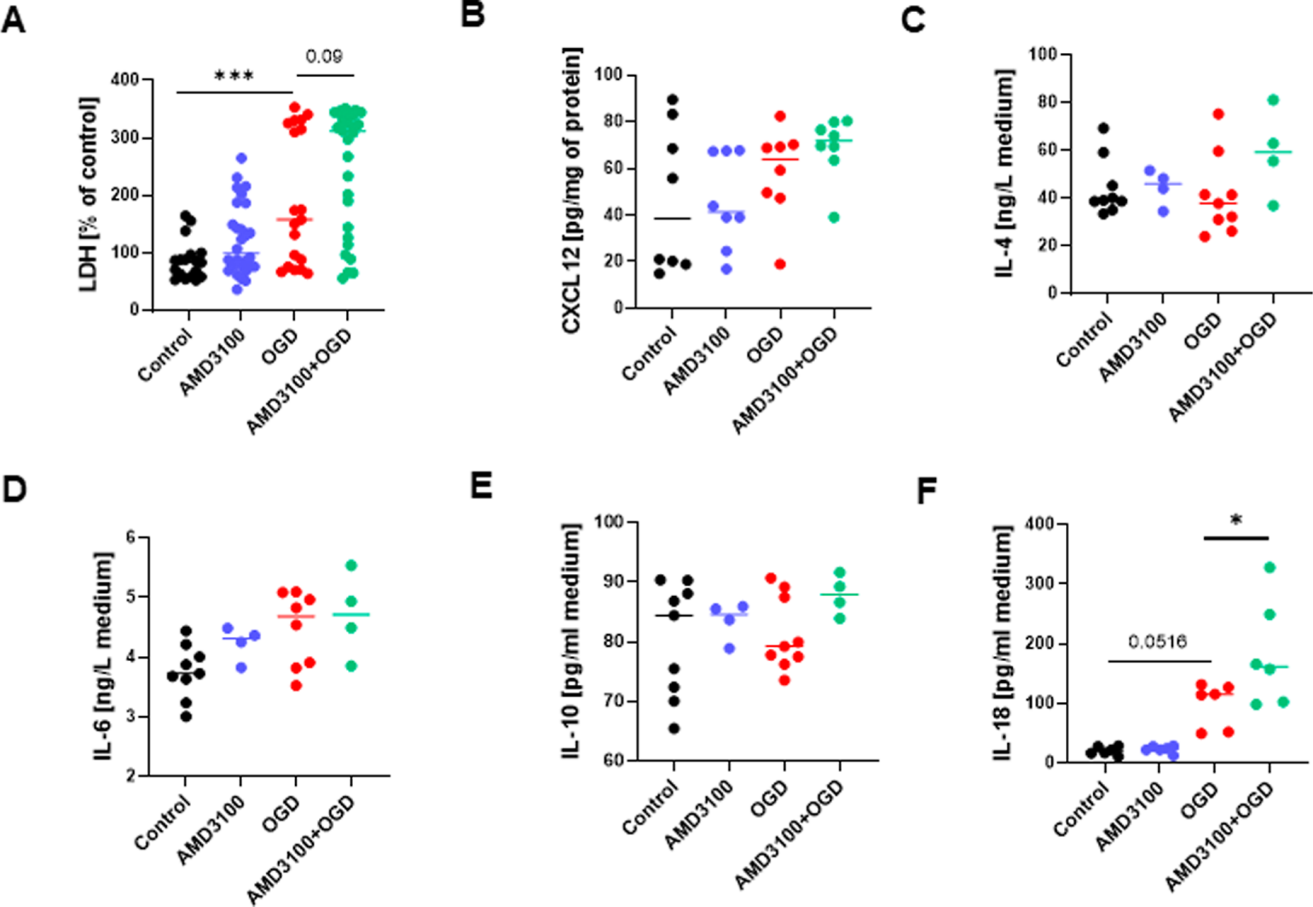
(A) Impact of OGD and CXCR4 modulation by AMD3100 on LDH release by OHC. Impact of AMD3100 and OGD on expression of (B) CXCL12, (C) IL-4, (D) IL-6, (E) IL-10, and (F) IL-18 by OHC.

### Modulation of CCR2 with Irbesartan Affects OHC Biological and Biophysical Response to OGD

We observed a significant increase in CCL2 expression following OGD (Figure 1D), suggesting that the CCL2-CCR2 axis may play a key role in the OHC response to OGD-induced stress. To verify this hypothesis, we administered the CCR2 receptor antagonist Irbesartan. While Irbesartan did not affect LDH release under control conditions, it significantly reduced LDH release in the OGD-subjected OHC (Figure 4A). Next, we investigated whether CCR2 modulation with Irbesartan influences the expression of pro- and anti-inflammatory cytokines relevant to stroke-induced neuroinflammation, namely IL-1β, IL-4, and IL-6. We found that Irbesartan did not affect IL-1β expression under control conditions, but significantly reduced its expression after OGD (Figure 4B). Interestingly, Irbesartan led to a significant reduction in IL-4 expression in control samples, but not in OGD-subjected OHCs (Figure 4C). No significant changes in IL-6 expression were observed in either condition (Figure 4D).

**Figure 4:**
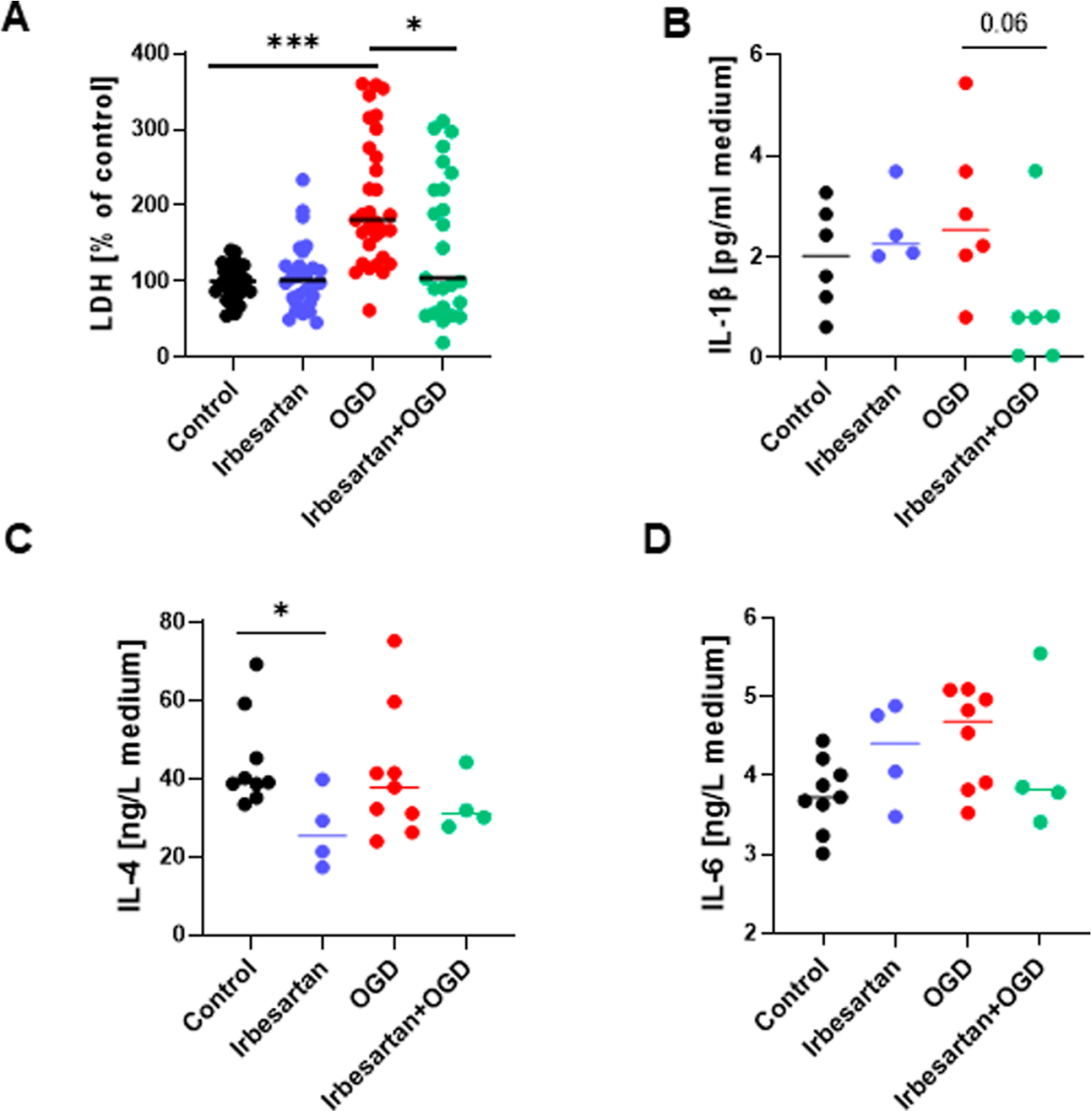
(A) Impact of OGD and CCR2 modulation by Irbesartan on LDH release by OHC. Impact of Irbesartan and OGD on expression of (B) IL-1β, (C) IL-4, and (D) IL-6 by OHC.

Previously, we showed that OGD leads to increased heterogeneity in mechanical properties and disrupts cytoskeletal organization in OHCs.^16^ Given that the immune response has been linked to alterations in the mechanical environment,^21,22,30^ we next aimed to investigate the impact of Irbesartan on the biomechanical response of OHCs to OGD.

Confocal imaging revealed that both Irbesartan and OGD influence the cytoskeletal organization of OHC (Figure 5). In control OHCs treated with Irbesartan, we observed an increased number of actin stress fibers compared to untreated controls (Figure 5). However, no major changes in microtubule organization were noted between control and Irbesartan-treated OHCs (Figure 5). In contrast, OGD led to pronounced disorganization of both the actin and microtubular cytoskeleton (Figure 5). Notably, in OHCs subjected to both OGD and Irbesartan treatment, actin organization resembled that of Irbesartan-treated controls, while microtubule disruption was less severe than in OGD-only samples (Figure 5).

**Figure 5:**
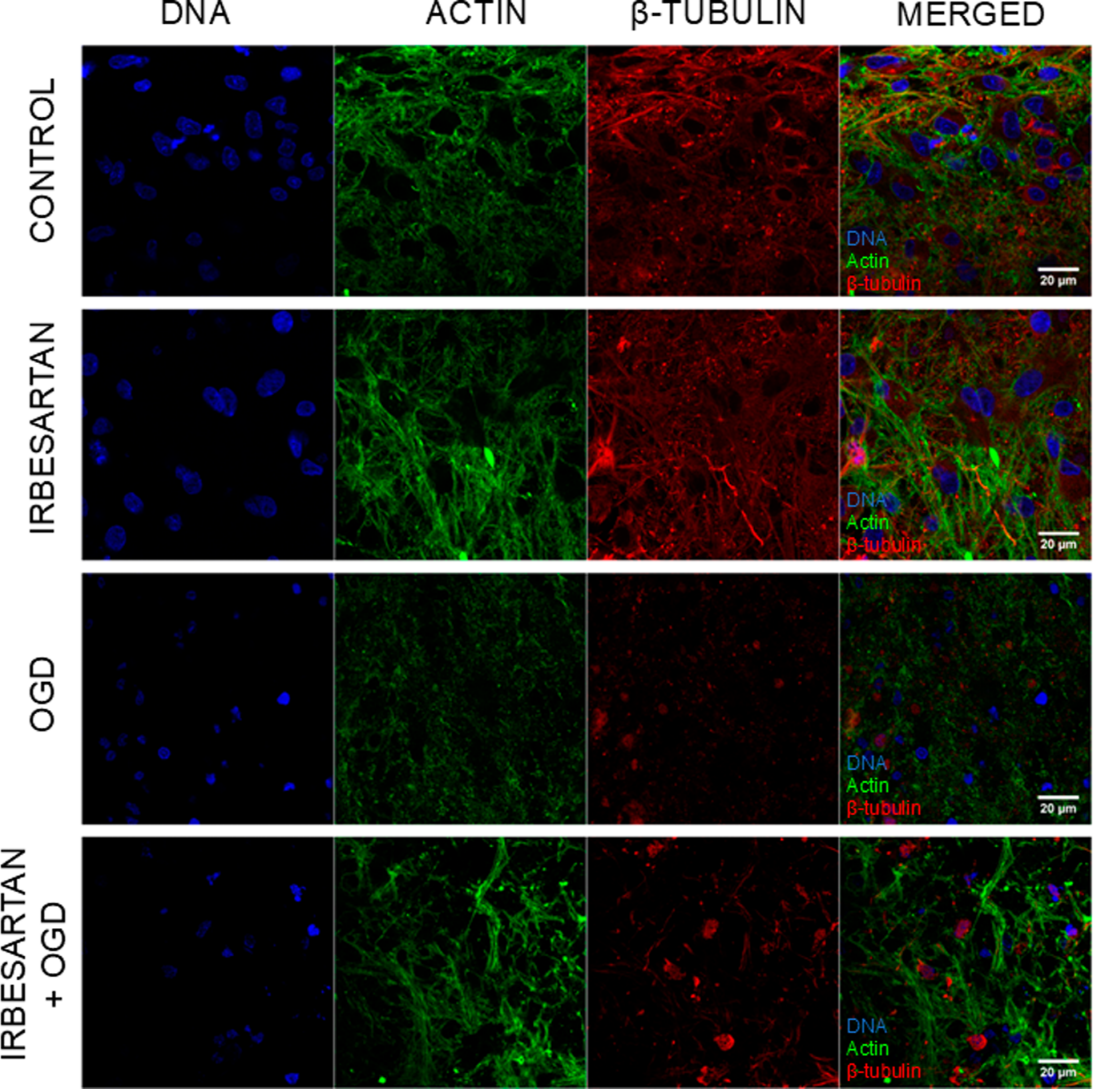
Immunofluorescence staining of OHC: nuclei (blue—DNA), actin cytoskeleton (green, phalloidin), microtubules (red—β-tubulin).

Given that we observed changes in cytoskeleton organization upon Irbesartan action, we next examined whether treatment with Irbesartan influences the mechanical properties of OHCs. Using atomic force microscopy working in a force spectroscopy mode, we measured elasticity within the CA1 region of OHCs (Figure 6A). The elastic (Young’s) modulus (E) was calculated from the force curves using the Hertz–Sneddon model^31^ (Figure 6B), and assessed as a function of indentation depth. In Irbesartan-treated control OHCs, the elastic modulus gradually decreased with increasing indentation depth (Figure 6C). In control, OGD, and Irbesartan + OGD groups, we observed the highest E value at ∼300 nm indentation, followed by a gradual decrease (Figure 6C). At shallow (100 nm) and intermediate (900 nm) indentation depths, only Irbesartan-treated OHCs showed a significant increase in tissue rigidity compared to untreated controls (Figure 6D,E). At a deeper indentation of 1500 nm, OHCs subjected to OGD alone significantly stiffened compared to controls (Figure 6F). Importantly, in OHCs treated with Irbesartan and subjected to OGD, the elastic modulus was partially restored to control levels for 1500 nm indentation depth (Figure 6F).

**Figure 6:**
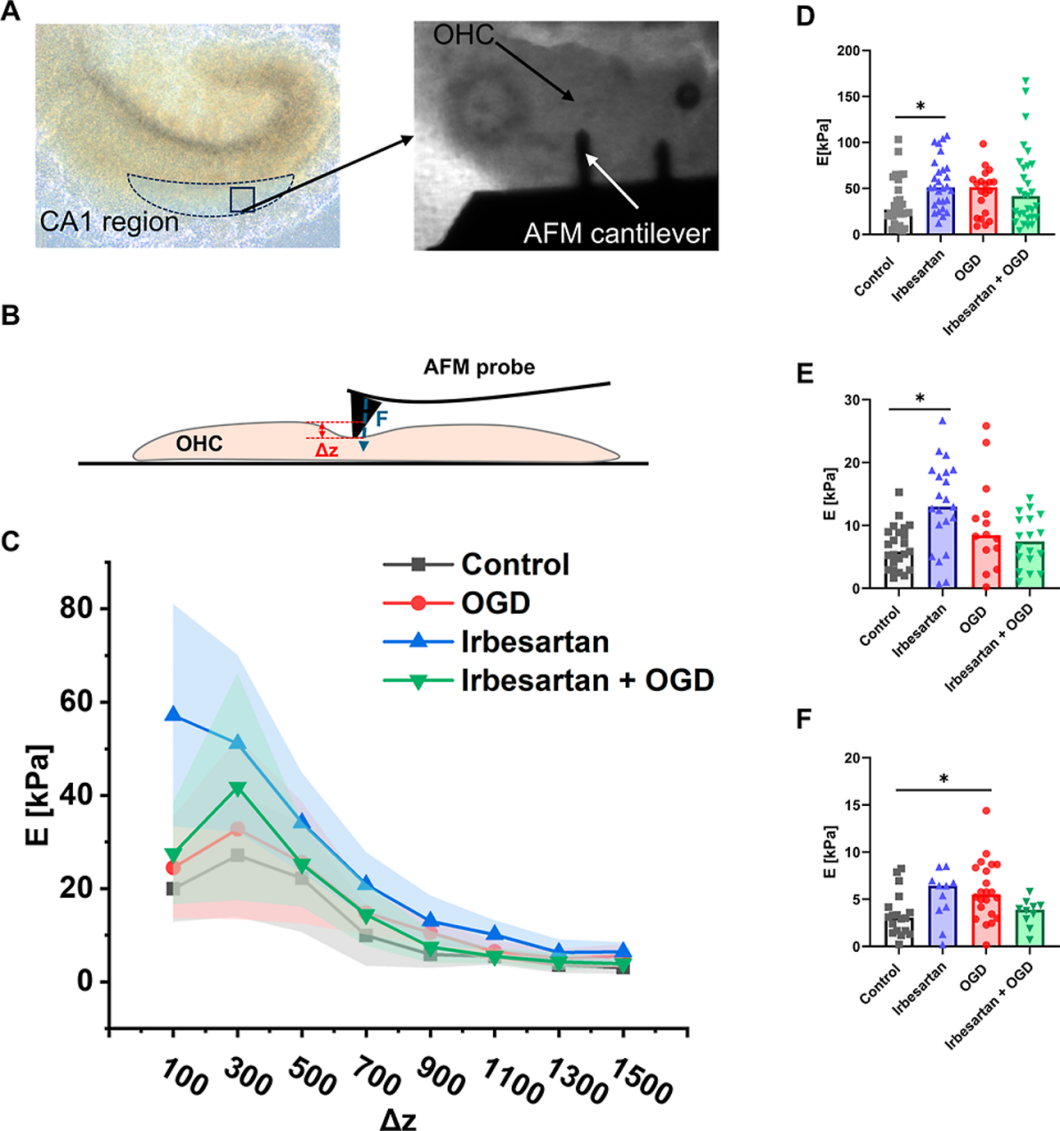
(A) Representation of the AFM experimental setup. Left: image of an entire OHC with the CA1 region marked. Right: view from an inverted microscope during AFM measurement. (B) Scheme of indentation of OHC. (C) Indentation-dependent variation in mechanical properties of OHC (mean ± SD shaded area plot). Elastic moduli of OHC for indentation of (D) 100 nm, (E) 900 nm, and (F) 1500 nm.

In summary, our results demonstrated that Irbesartan not only reduces cell death in OHCs subjected to OGD but also modulates their mechanical properties. This suggests that CCR2 antagonism may provide a dual benefit in ischemic injury by limiting inflammation-induced damage and preserving tissue biomechanics.

## Discussion

Consistent with our previous findings,^16^ we demonstrated that organotypic hippocampal cultures (OHCs) subjected to oxygen-glucose deprivation (OGD) enable the monitoring of changes in brain tissue architecture, mechanical properties, inflammatory responses, and the evaluation of potential therapeutic interventions, and as such represent a robust model of brain ischemia.

In this study, we found that OGD significantly increases the expression levels of CX3CL1 and CCL2 in OHCs, whereas CXCL12 expression remains unaffected. Ahn et al. investigated the temporal dynamics of CX3CL1 expression in the CA1 region of the gerbil hippocampus and observed that CX3CL1 immunoreactivity was elevated 24 h after ischemia.^32^ Since in our study we assessed CX3CL1 protein expression 24 h post-OGD and observed its upregulation, our results corroborate the relevance of the OHC model in recapitulating in vivo patterns of CX3CL1 regulation following ischemic injury. Moreover, the expression of the CX3CL1 receptor, CX3CR1, has been shown to increase following brain ischemia in multiple studies,^32,33^ underscoring the involvement of the CX3CL1-CX3CR1 axis in stroke pathophysiology. The clinical relevance of this axis is further supported by findings that elevated plasma CX3CL1 levels are associated with improved outcomes in stroke patients.^34^ Although the primary role of the CX3CL1-CX3CR1 axis in brain ischemia is believed to be modulating microglial activity, recent studies have presented conflicting evidence. Some reports suggest that activation of this axis exerts neuroprotective and anti-inflammatory effects postischemia,^35^ while others indicate that its inhibition improves recovery by attenuating neuroinflammation after both ischemic and hemorrhagic strokes.^36,37^ In our study, although microglia contributed to the neuroinflammatory response, pharmacological modulation of CX3CR1 with AZD8797 did not significantly impact OHC viability after OGD. These results further support the notion that the CX3CL1-CX3CR1 axis plays a context-dependent and time-sensitive role in ischemic injury, complicating its therapeutic targeting.^32^

Although CXCR4 expression was less pronounced than CX3CR1 expression, we nonetheless examined the CXCL12-CXCR4 axis. CXCR4 is expressed in microglia, and CXCL12-CXCR4 signaling has been shown to regulate neuronal development, axon branching, and morphogenesis.^38^ Moreover, CXCL12 influences GABA and glutamate release, ion channel function, and synaptic transmission.^11^ The clinical relevance of this pathway is highlighted by the availability of AMD3100 (Plerixafor), a CXCR4 antagonist approved by the U.S. Food and Drug Administration (FDA)^39^ and the European Medicines Agency (EMA).^40^ AMD3100 is clinically used for the mobilization of hematopoietic stem cells. In our model, AMD3100 induced a mildly proinflammatory response following OGD, consistent with reports that CXCL12 exerts neuroprotective effects that are inhibited by AMD3100.^41,42^ Conversely, other studies have reported beneficial effects of CXCR4 inhibition using AMD3100 ^43^ or CX549 ^44^ reflected by ameliorating neuroinflammation^43,44^ and reducing blood–brain barrier disruption.^43^

The CCL2-CCR2 axis is another key chemokine signaling pathway implicated in brain ischemia.^45^ CCL2 is recognized as a proinflammatory chemokine induced during immune activation and autoimmune responses.^46^ In agreement, we observed an elevated CCL2 expression in OHCs following OGD. Similar CCL2 increases have been reported in stroke models using primates,^47^ rats,^48,49^ and mice.^50,51^ Based on prior studies, we hypothesized that inhibition of the CCL2-CCR2 axis using Irbesartan would mitigate OHC death and inflammation following OGD. Irbesartan is a member of the Sartan family. We employed organotypic hippocampal cultures (OHCs) to minimize angiotensin II (AT1) receptor-dependent systemic effects of Irbesartan, such as alterations in blood pressure, vascular tone, and hormonal regulation,^52^ while preserving the physiological relevance of the tissue environment. This setup allowed us to isolate the direct effects of Irbesartan on CCR2, as the vessels within OHCs, although structurally present, are functionally disconnected from the circulation and thus unresponsive to systemic drug action.^53^ Indeed, we found that Irbesartan treatment reduced cell death and decreased the release of the proinflammatory cytokine IL-1β. Comparable results were obtained by He et al. (2019), who demonstrated that CCR2 inhibition using Propagermanium attenuated neuronal cell death and proinflammatory cytokines secretion (TNF-α, IFN-γ, IL-1β, IL-6, IL-12, IL-17, and IL-23) in a mouse model of middle cerebral artery occlusion (MCAO).^54^ Furthermore, Tsukuda et al. reported that coadministration of Irbesartan and Propagermanium enhanced the neuroprotective effect of CCR2 antagonism, as evidenced by reduced infarct volume following MCAO.^55^ Taken together, our study demonstrates that modulation of the CCL2-CCR2 axis with Irbesartan exerts a modest, but positive regulatory effect on OHCs. This is particularly relevant from a clinical perspective, as Irbesartan’s primary indication is the treatment of hypertension.^56^ Given that hypertension is a major risk factor for stroke,^9^ our findings shed new light on the potential neuroprotective role of Irbesartan in this patient population.

Previously, we reported that OGD induces stiffening of OHCs.^16^ We therefore investigated whether pharmacological modulation of CCR2 alters the biomechanical response of OHCs under ischemia-like conditions. To this end, we employed atomic force microscopy (AFM), a physical method that enables the determination of the mechanical properties of various biological samples, including hydrogels, cells, and tissues.^16,57^ While AFM has previously been used to study the mechanical properties of nervous tissues,^58^ to our knowledge, this is the first application of AFM to investigate the impact of a potential therapeutic agent on nervous tissue. We observed that Irbesartan treatment alone increased OHC rigidity, which was associated with cytoskeletal remodeling. Interestingly, cell stiffening was identified as a general stress response to various compounds, regardless of their specific mechanisms of action.^59,60^ Notably, we also detected a significant increase in OHCs rigidity following OGD, particularly at greater indentation depths. Fuhs et al. investigated brain stiffness ex vivo using an AFM in a rodent stroke model and similarly reported that the ischemic hemisphere exhibited increased rigidity compared to the contralateral side.^61^ Furthermore, local stiffening of brain tissue induced by solid tumors can exert mechanical stress on adjacent neurons and blood vessels, leading to vessel constriction and neuronal death.^62^ In line with this, targeting not only biological processes but also physical phenomena associated with disease represents a novel and promising therapeutic approach. Recent advances in mechanobiology have identified modulators of biomechanical pathways with potential for treating neurological disorders.^63^ Although targeting tissue mechanics remains at the preclinical stage,^64^ this emerging field provides valuable insights into drug action, as identifying biomechanical cues can inform candidate selection and guide eventual clinical evaluation.

Overall, our results highlight the multifaceted role of chemokine-receptor axes in shaping the neuroinflammatory response to stroke in the OGD–OHCs model. They further support the neuroprotective potential of Irbesartan as a modulator of the CCL2-CCR2 axis. One of the main challenges for its clinical application is Irbesartan’s limited ability to cross the blood–brain barrier (BBB).^65^ While this might initially appear as a limitation, our biomechanical analysis suggests a more nuanced interpretation. Under basal conditions, Irbesartan induced actin cytoskeletal remodeling and increased OHC stiffness, likely reflecting a mild, transient stress adaptation in otherwise healthy tissue. Given the minimal BBB penetration under physiological conditions, such effects are unlikely to occur in vivo. By contrast, following OGD-induced injury, Irbesartan normalized OHC mechanics, indicating a selective beneficial effect when neural tissue is mechanically and inflammation-compromised. Previous studies have shown that Irbesartan can support BBB repair after injury.^66^ Together with our data, this allows us to propose a self-limiting therapeutic mechanism: Irbesartan gains access to injured brain tissue through a transiently permeabilized BBB, restores cytoskeletal and mechanical homeostasis, and subsequently contributes to BBB regeneration, thereby limiting exposure to healthy tissue.

While this framework remains a proposed mechanistic model, it provides a biologically plausible explanation that integrates Irbesartan’s pharmacological profile with its context-dependent biomechanical effects. Our findings underscore that investigating tissue biomechanics not only deepens understanding of pathological mechanisms but also enables tracking of therapeutic effects.

## Materials and Methods

### Animals

Sprague–Dawley rats (weighing 200–250 g) were obtained from Charles River in Sulzfeld, Germany, and kept under standard conditions at 23 °C with a 12 h light/12 h dark cycle (lights on at 08:00), with free access to food and water. After a two-week quarantine and adaptation period, the estrous cycle phase of the females was identified. On the proestrus day, females were paired with males for 12 h, and the following morning, vaginal swabs were taken, confirming the presence of sperm. All experimental procedures followed the guidelines of the Committee for Laboratory Animal Welfare and Ethics of the Maj Institute of Pharmacology, Polish Academy of Sciences, Krakow, Poland. Efforts were made to reduce the number of animals used and minimize their distress.

### Organotypic Hippocampal Slices

As previously,^16^ hippocampi were isolated from 6 to 7-day-old rat pups. The hippocampi were then cut into 350 μm slices using a tissue chopper (McIlwain, TedPella, USA) and transferred to ThinCert membranes (Greiner Bio-One, Austria). Hippocampal slices were cultured for 7 days in a medium containing 50% DMEM + GlutaMax-I medium (Gibco, Great Britain) containing: 20% HBSS, 25% horse serum, 5 mg/mL glucose, 2% B-27 supplement, 2% fungisin, penicillin at a concentration of 100 U/ml and streptomycin at a concentration of 0.1 mg/mL.

The medium was changed every 2 days, gradually reducing the concentration of horse serum. On the sixth day of culture, the medium contained 50% DMEM F-12, 20% HBSS, 5 mg/mL glucose, 2% B-27 supplement, 2% N2 supplement, 2% fungisin, penicillin at a concentration of 100 U/ml, and streptomycin at a concentration of 0.1 mg/mL.

### Oxygen-Glucose Deprivation

On the seventh day of culture, oxygen and glucose deprivation were performed according to the protocol described in Bryniarska-Kubiak et al.^16^ Briefly, organotypic hippocampal cultures were rinsed twice in Ringer’s solution containing 10 mM mannitol (Sigma-Aldrich, Germany). Then, OHC was transferred to new 6-well plates containing Ringer’s buffer and placed in a hypoxic chamber (37 °C; gas flow: 95% N_2_, 5% CO_2_) for 40 min. After the OGD procedure, the insets with the slices were transferred back to a serum-free medium for 24 h under standard conditions (37 °C; 5% CO_2_).

### Microglia Silencing Procedure in Hippocampal Organotypic Cultures

To determine the role of the CX3CL1-CX3CR1 system in OGD processes, a procedure of “silencing” microglial cells that express CX3CR1 was performed in some hippocampal organotypic cultures using Clodronate (Merck Millipore, USA). On the day of establishing the culture, Clodronate was added to the culture medium at a final concentration of 10 μg/mL for 24 h. Then, the Clodronate medium was removed, and the hippocampi were further cultured under standard conditions.

### Modulation of Chemokine Receptors

To determine the role of chemokines (CX3CL1, CXCL12, CCL2) and/or their receptors (CX3CR1, CXCR4, CCR2, respectively) in the ischemia process, 1 h before the OGD procedure, the modulators (antagonists) were added to the organotypic cultures, appropriately for CX3CR1: AZD8797 (AxonMedChem, Nederlands)—0,05 mM, CXCR4: AMD3100 (Sigma-Aldrich, Germany)—1 μg/mL and CCR2: Irbesartan (Sigma-Aldrich, Germany)—0,1 mM.

After the OGD procedure, the cultures were transferred back to the medium containing modulators for 24 h. Then, their impact on the tested parameters was assessed in various experimental systems.

### Lactate Dehydrogenase Activity Test

The enzyme lactate dehydrogenase (LDH), which is released into the culture medium, was assessed 24 h after the OGD procedure and/or after incubation of organotypic cultures with modulators of individual chemokine receptors, or exogenous chemokine. As previously^50,67^ μL of medium was taken, and 50 μL of a mixture of reagents (consisting of a catalyst and a solution with a dye) provided by the kit manufacturer (Roche, Germany) was added, and then after 15–30 min. Incubation at 37 degrees, absorbance (wavelength λ = 490 nm) was measured using an automatic microplate reader Infinite M200 PRO (Tecan, Switzerland).

### ELISA Enzyme-Linked Immunosorbent Assays

Twenty-four h after the OGD procedure and/or after incubation of organotypic cultures with selected modulators of chemokine receptors, the following cytokines were determined using the ELISA assay: interleukin-6 (IL-6), TNF-α, IL-4, and IL-10 (Bioassay Technology Laboratory, UK) in the culture medium. The levels of chemokines: Chemokine C-X3-C-Motif Ligand 1 (CX3CL1) (Cloud Clone Corporation, USA), Stromal Derived Factor 1 (CXCL12/SDF-1) (Cusabio, USA) and Monocyte chemotactic protein 1 (CCL2/MCP-1) (Cusabio, USA) were determined in OHC homogenates, which were sonicated in RIPA buffer additionally containing: 1 mM sodium orthovanadate (Sigma, Germany); 1 mM PMSF (Sigma, Germany); 10 μg/mL protease inhibitors (P8340, Sigma, Germany) and 10 μg/mL phosphatase inhibitor mixture (P2850; P5726, Sigma, Germany). Then, the homogenates were shaken on ice for 30 min and centrifuged for 20 min. At 4 °C at 14,000 rpm. The obtained supernatants were collected and frozen at −80 °C. The total protein content in the supernatants was determined using bicinchoninic acid using the BCA method (ThermoScientific, United States). ELISA used commercially available analytical kits to determine the proteins mentioned above. Samples were loaded into 96-well plates in volumes according to individual manufacturers’ instructions. The concentration of samples was selected individually for each assay based on the test results performed at different dilutions. The detection limits were as follows: CX3CL1 < 0,055 ng/mg, CXCL12 < 0,195 pg/mg, CCL2 < 1.95 pg/mg, TNF-α < 2,51 ng/L, IL-6 < 0,052 ng/L, IL-4 < 0,46 ng/L, and IL-10 < 1.51pg/mL.

The intraseries errors of the determinations were less than 10%, and the interseries errors were less than 12%.

### Determination of Protein Levels Using the MILLIPLEX Method

Using the RECYTMAG-65KSTD Milliplex Map—Rat Cytokine/Chemokine Magnetic Bead Panel (Merck-Millipore, United States) method, the levels of cytokines IL-1β and IL-18 were assessed in hippocampal culture supernatants. Samples were loaded into 96-well plates in volumes according to the manufacturer’s instructions. The measurement was performed using an automatic MAGPIX reader (Luminex Corporation, United States), and the analysis was performed using xPONENT software (Luminex Corporation, United States). A subset of ELISA and MILLIPLEX data presented in this study, including the control and OGD-only conditions for the following cytokines: TNF-α, IL-1β, IL-4, IL-6, IL-10, and IL-18, was previously published in^16^ and is used here as a baseline for the treatment conditions. The control and OGD-only data included here originate from the same set of experiments as the treatment conditions examined in this study.

### Immunofluorescence Analysis

Immunofluorescence staining was performed in organotypic hippocampal cultures after the OGD procedure using the protocol established by Gogolla et al.^68^ OHC was fixed in 4% paraformaldehyde and treated with a 20% methanol solution. OHC were permeabilized with 0.05% Triton solution, and nonspecific bonds were blocked using 20% bovine serum albumin (BSA, Sigma, Germany) overnight at 4 °C. It was then incubated with the appropriate primary antibody: MAP-2 (1:300, Abcam, UK) IBA1 (1:200, Abcam, UK), β-tubulin-Cy3 (1:150, Sigma-Aldrich, Germany) overnight at 4 °C. OHC was incubated for 4 h at room temperature with the appropriate secondary antibody: goat antichicken AF488, donkey antigoat AF 555 (1:300, Abcam, UK). OHC was incubated for 1h at room temperature with Phalloidin-AF488 (1:200, Invitrogen, USA). In the final stage of the procedure, labeling with DAPI or Hoechst33342 dyes was performed to image cell nuclei. The preparations were visualized on a Leica TCS SP8 X fluorescence confocal microscope.

### Imagine Using Confocal Fluorescence Microscopy

The images were acquired using a Leica TCS SP8 X confocal laser scanning microscope (Leica Microsystems CMS GmbH, Mannheim, Germany) equipped with a 10× objective and a 63× oil immersion objective (HC PL APO CS2 1.40 NA OIL). Image acquisition was performed bidirectionally along the *X* axis at a scanning frequency of 200 Hz and an image format of 1648 × 1648 (pixel size 74 nm), digital magnification 1.5, and to reduce noise, images were recorded with a line average of 3 lines. The analysis was performed using ImageJ 1.53t software (Wayne Rasband, National Institute of Health, USA) with default parameters.

### Determination of Young’s Modulus of Hippocampal Organotypic Cultures Using Atomic Force Microscopy

The mechanical properties of organotypic hippocampal cultures were assessed using an atomic force microscope (AFM, XE-120, Park Systems, South Korea) operating in force spectroscopy mode. The measurements were conducted with an NSC36 C microbeam (μ-masch) featuring a nominal spring constant of *k* = 0.6 N/m and an opening half an gle of α = 20°. Before data acquisition, the photodetector sensitivity was calibrated by recording a force–distance curve on a nondeformable substrate (a glass coverslip).

For AFM measurements performed following the methodology described by Calò et al.,^58^ the cultures were fixed by incubating them in a 4% paraformaldehyde solution for 60 min. The membrane edge was then cut and affixed to a glass coverslip using a drop of cyanoacrylate adhesive to prevent contamination of the culture. During the measurements, the prepared samples were placed in liquid cuvettes filled with phosphate-buffered saline (PBS). To ensure accurate selection of the CA1 region of the hippocampus for mapping and to verify the correctness of flexibility measurements, an optical microscope integrated with the AFM was used in conjunction with topographic scanning of the examined area.

Force spectroscopy measurements were conducted on a 4 × 4 pixel grid, covering an area ranging from 10 × 10 μm to 26.7 × 26.7 μm. The maximum applied force during spectroscopic measurements was *F* = 15 nN, while surface topography was recorded using a lower force of 1 nN.

The acquired force–distance curves were converted into force–indentation relationships to quantify material deformation. Young’s modulus was then determined using the Hertz model, which describes the elastic deformation of two perfectly elastic bodies. The AFM probe shape was modeled according to Sneddon’s equations.^31^

### Young’s Modulus for a Cone-Shaped Probe was Calculated Using the Equation

F(Δz)=((2·tan(α)·E)/π)·(Δz)2

where *F* is the applied force, Δ*z* represents the indentation depth, α is the half-opening angle of the NSC36 C microbeam cone, and *E* is Young’s modulus.

Atomic force microscopy (AFM) measurements of tissue stiffness under control and OGD conditions were previously collected and partially presented in,^16^ where the focus was limited to the mechanical characterization of OGD-induced changes. In the present study, these AFM data sets were reanalyzed in combination with additional conditions, including treatment with Irbesartan obtained within the same experimental series. The entire data set was reprocessed using updated analytical protocols to enable direct comparison across all conditions.

### Statistical Analysis

All statistical analyses were performed using Origin 2023b and GraphPad. The Grubbs test was used to identify extreme values. Normality of distributions was assessed with the Shapiro–Wilk test, and the homogeneity of variances with the Levene test. The significance of differences between the compared groups was assessed using the Student’s *t*-test or the Mann–Whitney *U* test and one- or multiway analysis of variance (ANOVA). Data obtained in the LDH assay were normalized to the absorbance value of control cells (100%) and are shown as a percentage of control ± median. The results obtained from determinations using ELISA and MILLIPLEX methods were presented as ng or pg/mg of protein and ng or pg/mL of culture medium, while the results obtained from determinations using an atomic force microscope were expressed as kPa (median value of Young’s moduli ± quartile range). The following value was considered statistically significant: * *p* < 0.05, ** *p* < 0.01, and ***p < 0.001.

## Supplementary Material





## Notes

Immunofluorescence images of organotypic hippocampal cultures stained for neuronal (MAP-2) and microglial (IBA1) markers (PDF)
Quantitative analysis of mean fluorescence intensity (MFI) for MAP-2 and IBA1 (PDF)
