# Supplementary material for: Chemokine-Receptor Modulation Shapes Neuroinflammatory and Biomechanical Responses in Organotypic Hippocampal Cultures After Oxygen-Glucose Deprivation
Source: ACS Chem Neurosci. 2026 May 15;17(11):2096–107. doi: 10.1021/acschemneuro.6c00005 (PMC13318064; doi:10.1021/acschemneuro.6c00005)
Supplement: Supplementary file 2 [file cn6c00005_si_002.pdf]

# Supplementary material

## Chemokine-Receptor Modulation Shapes Neuroinflammatory and Biomechanical Responses in Organotypic Hippocampal Cultures After Oxygen-Glucose Deprivation

Natalia Bryniarska-Kubiak[1,2,*], Andrzej Kubiak[2,3], Ewa Trojan[1], Alessandro Podestà[4], Małgorzata Lekka[3], Agnieszka Basta-Kaim[1,*]

[1]Laboratory of Immunoendocrinology, Department of Experimental Neuroendocrinology, Maj Institute of Pharmacology, Polish Academy of Sciences, 31-343 Kraków, Poland

[2]Eli and Edythe Broad CIRM Center for Regenerative Medicine and Stem Cell Research, Keck School of Medicine, University of Southern California, Los Angeles, California 90033, United States

[3]Department of Biophysical Microstructures, Institute of Nuclear Physics, Polish Academy of Sciences, 31-342 Kraków, Poland

[4] Department of Physics "Aldo Pontremoli", Università degli Studi di Milano, via G. Celoria 16, 20133, Milano, Italy

Correspondence should be addressed to: Natalia.Bryniarskakubiak@med.usc.edu, basta@if-pan.krakow.pl

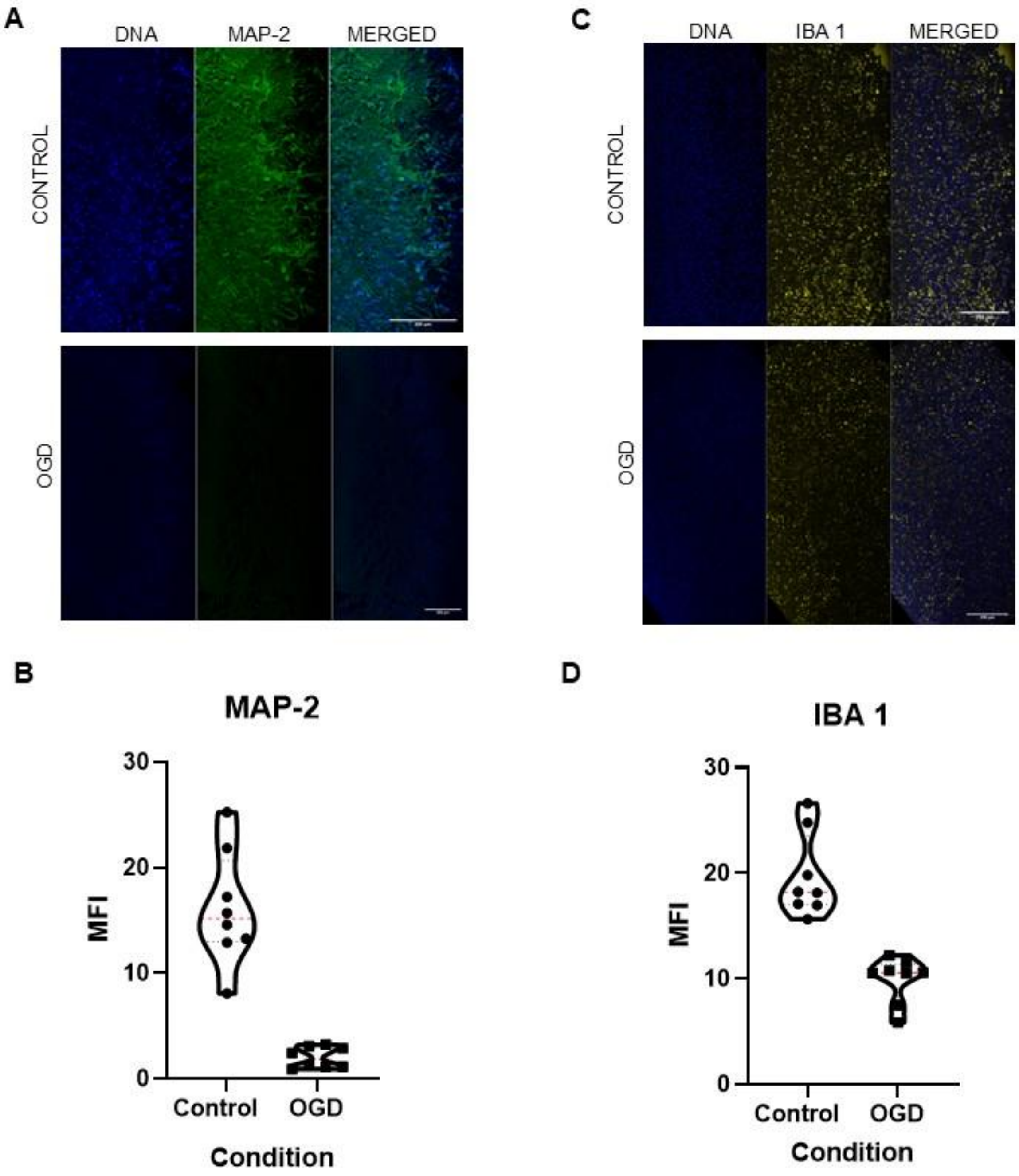


SI: Fig. S1

A) Immunofluorescence images of OHCs. Blue - DNA, green - neuronal marker MAP-2.

B) Mean fluorescence intensity (MFI) of OHCs stained with MAP-2 antibody; each dot represents one image analyzed.

C) Immunofluorescent images of OHCs. Blue - DNA, yellow - microglial marker IBA1.

D) Mean fluorescence intensity (MFI) of OHCs stained with IBA1 antibody; each dot represents one image analyzed.
